# Time-Domain Microfluidic Fluorescence Lifetime Flow Cytometry for High-Throughput Förster Resonance Energy Transfer Screening

**DOI:** 10.1002/cyto.a.22616

**Published:** 2014-12-18

**Authors:** Jakub Nedbal, Viput Visitkul, Elena Ortiz-Zapater, Gregory Weitsman, Prabhjoat Chana, Daniel R Matthews, Tony Ng, Simon M Ameer-Beg

**Affiliations:** 1 Division of Cancer Studies, King's College LondonUnited Kingdom; 2 Randall Division of Cell and Molecular Biophysics, King's College LondonUnited Kingdom; 3 Division of Asthma, Allergy & Lung Biology, King's College LondonUnited Kingdom; 4 Immune Monitoring Laboratory, NIHR Biomedical Research Centre at Guy's and St Thomas' NHS Foundation Trust and King's College LondonUnited Kingdom; 5 Queensland Brain Institute, The University of QueenslandSt Lucia, Australia; 6 UCL Cancer Institute, University College LondonUnited Kingdom

**Keywords:** flow cytometry, fluorescence lifetime, microfluidics, time-correlated single photon counting, FRET

## Abstract

Sensing ion or ligand concentrations, physico-chemical conditions, and molecular dimerization or conformation change is possible by assays involving fluorescent lifetime imaging. The inherent low throughput of imaging impedes rigorous statistical data analysis on large cell numbers. We address this limitation by developing a fluorescence lifetime-measuring flow cytometer for fast fluorescence lifetime quantification in living or fixed cell populations. The instrument combines a time-correlated single photon counting epifluorescent microscope with microfluidics cell-handling system. The associated computer software performs burst integrated fluorescence lifetime analysis to assign fluorescence lifetime, intensity, and burst duration to each passing cell. The maximum safe throughput of the instrument reaches 3,000 particles per minute. Living cells expressing spectroscopic rulers of varying peptide lengths were distinguishable by Förster resonant energy transfer measured by donor fluorescence lifetime. An epidermal growth factor (EGF)-stimulation assay demonstrated the technique's capacity to selectively quantify EGF receptor phosphorylation in cells, which was impossible by measuring sensitized emission on a standard flow cytometer. Dual-color fluorescence lifetime detection and cell-specific chemical environment sensing were exemplified using di-4-ANEPPDHQ, a lipophilic environmentally sensitive dye that exhibits changes in its fluorescence lifetime as a function of membrane lipid order. To our knowledge, this instrument opens new applications in flow cytometry which were unavailable due to technological limitations of previously reported fluorescent lifetime flow cytometers. The presented technique is sensitive to lifetimes of most popular fluorophores in the 0.5–5 ns range including fluorescent proteins and is capable of detecting multi-exponential fluorescence lifetime decays. This instrument vastly enhances the throughput of experiments involving fluorescence lifetime measurements, thereby providing statistically significant quantitative data for analysis of large cell populations. © 2014 International Society for Advancement of Cytometry

## Introduction

Flow cytometry is a cell biology technique broadly applied in the life science research and the clinic. The most common readouts in flow cytometry are forward- and side-scattering properties of particles and fluorescence intensities of attached probes measured in multiple spectral channels. Specialized instruments record spectrally resolved fluorescence intensity, rare-earth metal atom counts, fluorescence intensity images, fluorescence anisotropy, or fluorescence lifetime to extract additional information from the sample (1–15). We present a fluorescence lifetime measuring flow cytometer for quantification of biomolecular interactions by means of Förster resonance energy transfer (FRET) or sensing of local fluorophore environment. The technique positions itself between fluorescence lifetime imaging (FLIM) microscopy and flow cytometry by providing higher cell-throughput at the expense of spatial information while offering the ability to extract fluorescence lifetime information.

The fundamental photo-physical parameter of fluorescence lifetime is used broadly in microscopy of cells and tissues for quantitative measurements of biomolecular interaction by FRET and for molecular environment sensing (16–18). Despite the development of a number of instruments offering fluorescence lifetime detection in flow cytometry, predominantly in the frequency domain (9–15) but also in the time-domain (19,20), this remains a niche methodology. In part, this may be due to the inherent challenge of measuring fluorescence lifetime from a limited number of photons emitted in the short time taken for the particle or cell to pass through the measurement volume of the flow cytometer. An additional obstacle is the considerable complexity in the implementation of fluorescence lifetime-measuring methods. In this article, we present the development of a microfluidic fluorescence lifetime flow cytometer. It is based on burst integrated fluorescence lifetime (BIFL) detection, a principle well established in the field of single-molecule spectroscopy (21–23). In brief, a single fluorescent molecule diffusing through a confocal volume generates a burst of photons, which are detected with a photomultiplier and the fluorescence decay is measured using time-correlated single photon counting (TCSPC). In our implementation, single molecules are replaced by suspension cells pumped through a microfluidic chip. In particular, we demonstrate that fluorescence lifetime measurement in our system can report on cell membrane lipid order, FRET in biosensors, and tyrosine phosphorylation in epidermal growth factor (EGF) receptor.

FLIM, in which image contrast is afforded by spatial variation in fluorescence lifetime of the probe, has established its place in the life sciences for quantitative imaging (17,24,25). Contrast is largely concentration and intensity independent but sensitive to the solvent environment of the fluorophore, for example pH (26), Ca^2+^ concentration (27), protein-bound NADH fraction (28), refractive index (29), or viscosity (30). Measurement of the near-field localization of protein complexes may be achieved by the detection of FRET between fluorophore-conjugated proteins (31). FRET is a non-radiative, dipole–dipole coupling process whereby energy from an excited donor fluorophore is transferred to an acceptor fluorophore in close proximity (32,33). Since the process depletes the excited state population of the donor, FRET will both reduce the fluorescence intensity and the donor fluorescence lifetime. The major advantage of using donor FLIM to detect FRET is that the method is independent of fluorophore concentration, donor–acceptor stoichiometry, and light path length and is therefore well suited to studies on intact cells (25,34–43). The FRET/FLIM technique for probing protein–protein interactions is well established in research on cell signaling pathways (35,39,40,44–47). We, and others, have adapted FLIM-based protein–protein interaction assays to directly monitor post-translational modifications of proteins within live or fixed cells. Examples include phosphorylation of protein kinase C α (34), Akt/PBK (48) or EGF receptor (49–51), ubiquitination (52), and sumoylation of protein-tyrosine phosphatase 1B (53). Given the prevalence for fluorescent lifetime approaches in imaging of FRET interactions for quantitative analysis, it is important to note that fluorescence lifetime flow cytometry has not, to our knowledge, been used to investigate protein interactions in cell populations by FRET.

Flow cytometric FRET quantification on a cell-by-cell basis has typically applied ratiometric methodology (54–57) or fluorescence anisotropy measurements (3–6). Ratiometric techniques permit quantification of FRET based on the fluorescence intensity signals recovered in the donor and acceptor spectral channels. Correct estimation of FRET, however, requires meeting a series of assumptions imposed by the mathematical model. These might be difficult or impossible to fulfill and often limit flow cytometry FRET assays to applications with biosensors featuring intra-molecularly consistent fluorophore stoichiometry and carefully controlled experiments. Often FRET detection in flow cytometry is simply reduced to qualitative, rather than quantitative measurements. Quantification based on changes in fluorescence anisotropy applies sophisticated models of energy transfer that require deep understanding of the underlying photo-physics particular to each experiment. In flow cytometry, homoFRET, occurring between spectrally identical fluorophores, has been demonstrated on a customized flow cytometer equipped with polarization sensitive detectors (3–5). HeteroFRET, occurring between two spectrally distinct fluorophores, has been detected as the increase in fluorescence anisotropy of the donor fluorophore (58,59). With improved dynamic range, heteroFRET can also be sensed through a reduction in the acceptor fluorescence anisotropy (6,60,61). While ratiometric and polarization anisotropy flow cytometry techniques can, in principle, recover quantitative information on FRET, they remain limited to particular applications and experimental conditions.

Recently, Houston et al. (13) presented a flow cytometer for sorting of cells on the basis of fluorescence lifetime using a digital multi-frequency methodology in a modified commercial flow cytometer. The methodology is most robust when the fluorescence lifetimes of dyes used vary considerably (4–5 ns difference in the example shown in Ref.(13)). Since measurement errors are large, small changes in fluorescence lifetime as measured in FRET by donor quenching are likely to be lost in the noise. This is of particular relevance when considering fluorescence lifetime FRET measurements since small lifetime changes (of the order of 100 ps) are common. Appropriate signal processing of raw data from an unmodified state-of-the art flow cytometer can yield a fluorescence lifetime estimate based on the delay between the measured fluorescence and scatter response to passing cells (62). While this technique does not require any specialized hardware, the associated measurement errors limit its fluorescent lifetime resolution to the order of nanoseconds.

In the present study, we describe the development, characterization, and applications of a flow cytometer with time-resolved fluorescence lifetime measuring capability. The instrument combines pulsed laser excitation and TCSPC detection on an epifluorescence microscope. A microfluidics device pumps cell suspension through the microscope objective's focal volume. Subsequent computer software analysis yields multi-channel data resembling those of a commercial flow cytometer. Fluorescence lifetime, fluorescence intensity, mean photon count rate, and fluorescence burst duration are assigned to each particle. The optimal operating condition ranges were established with the maximum throughput reaching 3,000 particles per minute. The instrument vastly enhanced the throughput of experiments depending on fluorescence lifetime measurements, thereby providing data on an unprecedented number of individual cells.

The instrument has been applied in three assays with mammalian cells. Cell-specific changes in local membrane organization were measured using di-4-ANEPPDHQ (1-[2-hydroxy-3-(N,N-di-methyl-N-hydroxyethyl)ammoniopropyl]-4-[β-[2-(di-n-butylamino)-6-napthyl] vinyl]pyridinium dibromide), a lipophilic environmentally sensitive dye that exhibits changes in its fluorescence lifetime as a function of membrane lipid order. Donor fluorescence lifetime and FRET efficiency were measured in live cells expressing three different FRET standards, which are chimeric proteins consisting of green fluorescent protein (GFP) and red fluorescent protein (RFP) separated by different peptide linkers. Finally, an EGF stimulation assay demonstrated the instrument's capacity to separate the kinase activity on epidermal growth factor receptor (EGFR, ErbB1, HER1) from other target proteins. To the best of our knowledge this is the first report of FRET measured using fluorescence lifetime flow cytometry.

EGFR belongs to the family of ErbB receptors involved in transduction of extracellular proliferative signals (63) resulting in cell proliferation, motility, and resistance to cytotoxic agents. EGF binding results in receptor homo- or hetero-dimerization with other members of the family, followed by tyrosine phosphorylation (pY) in the intracellular domain of the receptor (64). This attracts adapter proteins leading to the formation of a signaling complex and activation of downstream signaling molecules like Akt/PKB and ERK1/2. Expression of EGFR was found to be a strong prognostic factor for head and neck, ovarian, cervical, bladder, and esophageal cancers (65). However, it is the phosphorylated state of EGFR that is pathologically significant. Inhibitors have been developed to target EGFR tyrosine phosphorylation, or downstream targets, to overcome developed resistance to anti-EGFR inhibitors (66). The ability to detect phosphorylated EGFR in tumor cells, especially in tumor circulating cells, may provide an additional insight into the biology of cells with greater metastatic potential. We demonstrate that phosphorylated EGFR is detectable by fluorescence lifetime flow cytometry utilizing a FRET assay adapted from Ref.(49).

## Materials and Methods

### Instrument

#### Time-correlated single photon counting microscope

**Figure 1:**
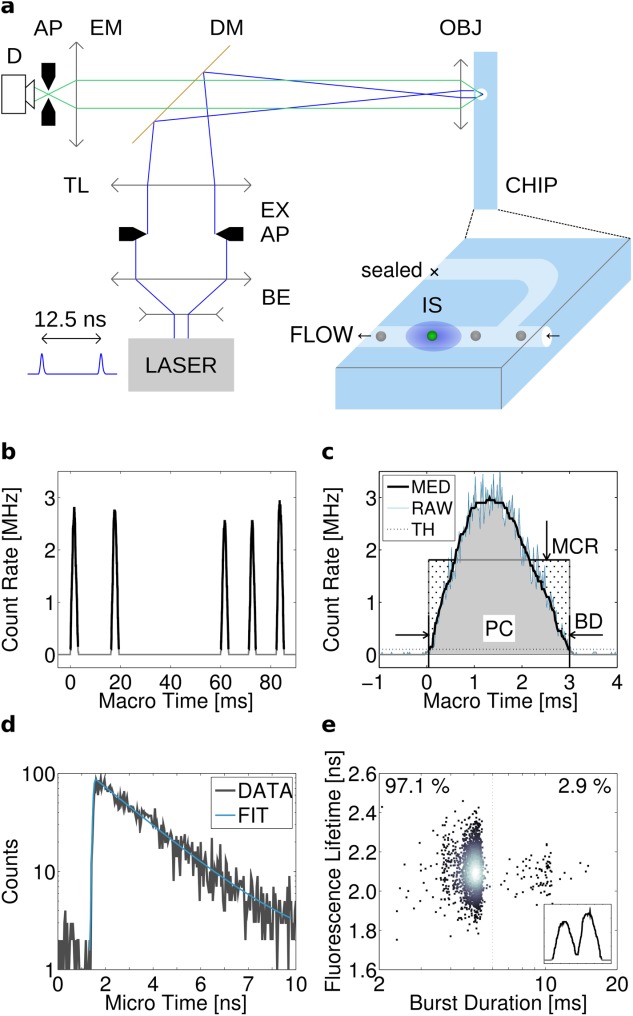
Experimental setup and data analysis. (a) A microfluidics chip (CHIP) flow channel was positioned in the focus of a widefield epi-fluorescence microscope objective (OBJ). A circular illumination patch (IS) intersects flowing particles exciting fluorescence. The microscope features a picosecond pulsed laser (LASER), beam expander (BE), variable field aperture (AP), excitation filter (EX), tube lens (TL), dichroic mirror (DM), emission filter set (EM), and a detector (D), converting photons into electrical signals for TCSPC. (b) Train of detected photons is segmented into single-particle bursts. (c) Single-particle bursts are extracted by step-wise histogramming of photon arrival macro times into 20 μs time bins (RAW, blue line), median filtering (MED, black line), and applying minimum threshold (TH, dotted line). Bursts are characterized by burst duration (BD), photon count (PC), and mean count rate (MCR). (d) Fluorescence lifetime of each particle is obtained by fitting an exponential decay model into transients created by binning micro times of photons contained in each burst. (e) Burst parameters are plotted into 2D scatter plots used in flow cytometry analysis. 2.9% bursts result from the improper segmentation of two-particle events (inset). [Color figure can be viewed in the online issue, which is available at http://wileyonlinelibrary.com.]

Measurements were performed on a modified automated microscope, developed in-house (61,67). The microscope (Fig. 1a) was equipped with a ×20 0.5 NA air objective (Nikon Instruments Ltd, Kingston Upon Thames, UK). A 473 nm pulsed diode laser (Becker & Hickl GmbH, Berlin, Germany) operated at 80 MHz was used for wide-field epifluorescent illumination of a circular area of 120 μm diameter. The filter set consisted of a 470 ± 11 nm single band-pass excitation filter (part FF01–470/22–25, Semrock, Rochester, NY), a FITC/TRITC dual band-pass dichroic mirror (part 59004, Chroma Inc, Olching, Germany), 520 ± 30 nm single band-pass excitation filter (part FF01–520/60-25, Semrock), and 488 nm long-pass filter (part LP02–488RE-25, Semrock). For dual-color detection, a 560 nm dichromatic mirror (part T560lpxr, Chroma) was added to split the emission light. The red part of the spectrum was further selected by 610 ± 37.5 nm emission filter (part HQ610/75, Chroma) and a 561 nm long-pass filter (part LP02–561RE-25, Semrock). The excitation source power was regulated by a series of absorptive ND filters in a motorized filter wheel (Thorlabs UK Ltd, Ely, UK). The filtered fluorescence light was relayed onto two hybrid photomultiplier detectors (part HPM-100-40, Becker & Hickl GmbH). The appropriate detector was connected to a TCSPC module (SPC-830, Becker & Hickl GmbH) and a control module (DCC-100, Becker & Hickl GmbH). In experiments requiring acquisition in two spectral channels, a detector router (HRT-41, Becker & Hickl GmbH) was connected between the two detectors and the TCSPC card. Microscope adjustment and acquisition were controlled by the Gray Institute Open Microscope control software (67), SPCM and DCC-100 programs (Becker & Hickl GmbH).

#### Microfluidics

All microfluidics components were sourced from Dolomite Microfluidics (Royston, UK). A pressure pump (Mitos P-Pump) was used to pump cell suspension into the inlet of a glass microfluidics T-Junction chip. The straight outlet from the chip was connected to a Mitos flow rate sensor before collecting or discarding the measured cell suspension. The perpendicular T-junction chip outlet was sealed off to prevent formation of cell clumps at the T-junction. All fluidic connections were facilitated by fluorinated ethylene propylene 0.25-mm internal diameter tubing. The chip was secured inside the microscope stage insert (Nikon Instruments) using a custom-designed plastic holder (Supporting Information Note 3) produced in-house by fused deposition modeling on a Dimension SST768 3D Printer (Stratsys, Derby, UK).

### Measurement

The measurement principle closely resembled standard flow cytometry technique. Fluorescent particle suspensions were pumped through a microfluidic chip channel intersecting a measurement volume located above the microscope objective. The fluorescent photon flux was measured and used to detect passing cells and infer their fluorescent lifetime, similarly to BIFL assays known from single-molecule spectroscopy (21–23).

#### Data acquisition

The fluorescence photons emitted from passing particles were detected by a photon counting detector linked to a TCSPC card with its accompanying software (see Fig. 1a and Instrument section above). The TCSPC system was used in first-in first-out (FIFO) mode that records a stamp including macro- and micro time for each detected photon (68). The macro time refers to the photon arrival time in the lab-frame of reference. Macro time can span the total measurement time, up to tens of minutes with microsecond resolution, depending on the duration of the experiment. The macro times are used to extract bursts of photons (500–10,000) originating from individual passing particles (Figs. 1b and 1c). The micro time defines the delay between the fluorescence photon emission event and the laser synchronization pulse and ranges between 0 and 10 ns. The raw data set size typically ranges between 300 and 800 kilobytes for each second of acquisition, depending on the number of passing particles and their brightness. The capacity to record the data is only limited by the size of the available disk storage space.

#### Burst integrated fluorescence lifetime analysis

The fluorescence lifetime of a passing particle was obtained by fitting an exponential function to the histogram of micro times of photons originating in one burst (Fig. 1c). Detailed description is included in Supporting Information Notes 1 and 2. In brief, the resulting datasets were analyzed by custom-written Matlab (Mathworks, Cambridge, UK) software (https://github.com/jnedbal/bifl). The software retrieved bursts of photons from individual passing fluorescent particles. This was achieved by distributing all recorded photon macro times into 20 μs histogram bins spanning the entire duration of the experiment. An adjustable threshold then segmented a median-filtered histogram of photon density into separate photon bursts (Fig. 1c). Each burst was characterized by its mean photon count rate, burst duration, and photon count. The average fluorescent lifetime for each burst was calculated using a Bayesian algorithm or Levenberg–Marquardt bi-exponential global analysis algorithm in the TRI2 fluorescence lifetime fitting software (69,70) (Fig. 1d). The results from fluorescence lifetime analysis were utilized in one- or two-dimensional flow cytometry scatter plots (Fig. 1e).

#### Sample handling and instrument calibration

Cells or bead suspensions were studied at concentrations below 3 × 10^5^ ml^−1^ to ensure sufficient spatial separation in the microfluidics channel and predominantly mono-particulate bursts. Cell or bead suspensions were pumped by an overpressure of 20 kPa. The suspension was mixed by a magnetic stirrer for 2 sec once every 3 min to avoid aggregation and sedimentation. After the individual experimental runs, the microfluidics system was cleaned by pumping 1 M NaOH followed by deionized water. Prior to each experiment, the chip position and objective focus were adjusted on a sample of 10 μm G1000 fluorescent micro-particles (Fisher Scientific, Loughborough, UK) to ensure the highest burst integrated fluorescence intensity and the most symmetrical burst profile.

#### Conventional flow cytometry with di-4-ANEPPDHQ

Conventional flow cytometry of cells stained with di-4-ANEPPDHQ was performed on FACSCalibur™ flow cytometer (BD Biosciences, Oxford, UK). Fluorescence intensities were recorded at 530 nm for GFP and at 585 nm for RFP with common excitation at 488 nm. Analysis was performed with custom-written Matlab scripts.

#### FRET standards in conventional flow cytometry

FRET efficiency ratiometric quantification was performed in accordance to the protocol outlined in Ref.(57) on cells analyzed with LSRFortessa™ flow cytometer (BD Biosciences). Fluorescence intensity was detected in three channels using two excitation lasers for cells transfected with either one of the three FRET standards: a GFP only, RFP only, or an empty vector. Directly excited donor emission channel utilized 488 nm laser excitation and 510 ± 7.5 nm detection. Sensitized acceptor emission channel combined 488 nm laser excitation with 585 ± 21 nm detection. Directly excited acceptor emission channel signal was measured with a 561 nm excitation laser and 582 ± 7.5 nm detection. See the MIFlowCyt item checklist in the Supplementary Information for further details.

### Cellular Experiments

#### Cell culture conditions

Human embryonic kidney 293 T-antigen cells (HEK293T), human epidermoid carcinoma skin cells (A-431), and Michigan Cancer Foundation-7 (MCF-7) (all American Type Culture Collection) cells were cultured in RPMI-1640, DMEM, and DMEM, respectively, supplemented with 10% fetal calf serum, l-glutamine, penicillin, and streptomycin in an 5% CO_2_-enriched atmosphere at 37°C.

#### FRET standards

GFP-RFP FRET standards (61), C-RFP (61), pEGFP-C3 (Clontech, Mountain view, CA) and pCDNA3.1-MH-A(+) (Life Technologies, Paisley, UK) were transfected using jetPEI® (Polyplus-transfection, Illkirch, France) according to manufacturer's instruction into HEK293T cells 24 h prior to analysis. Cells were rinsed with phosphate buffered saline (PBS), detached with Trypsin/EDTA (disodium ethylenediaminetetraacetate) and re-suspended in Opti-MEM® (Life Technologies) with 25 mM HEPES (4-(2-hydroxyethyl)piperazine-1-ethanesulfonic acid) for analysis.

#### EGFR tyrosine phosphorylation

Approximately, 2 × 10^6^ MCF-7 cells were plated into 100-mm diameter culture dishes. Twenty-four hours later EGFR-GFP (71) and pEGFP-C3 (Clontech) were transfected using FuGENE® 6 (Promega, Southampton, UK) according to manufacturer's instructions. After 24 h, cells were rinsed with PBS and lifted off the dish during a 10-min incubation with Accutase (Sigma-Aldrich, Gillingham, UK) at 37°C. Cells were carefully triturated three times using a 19G needle and a 20 ml hypodermic syringe prior to pelleting and re-suspending in culture medium at a concentration of 10^6^ ml^−1^. Optional recombinant human EGF (Peprotech, London, UK) was added to the cells at 100 ng ml^−1^ final concentration and incubated at 37°C for 5 min. Cells were fixed by adding an equal volume of freshly prepared 4% paraformaldehyde (Thermo Scientific Ltd, Cramlington, UK) in PBS for 10 min, followed by a 20-min permeabilization on ice with 0.1% Triton-X (Sigma-Aldrich) in PBS, two 10-min treatments with sodium borohydrate (1 mg ml^−1^, Sigma-Aldrich) in Tris buffered saline with 2 mM EDTA (TBSE, Sigma-Aldrich). Cells were blocked for 15 min in a 1% bovine serum albumin (GE Healthcare, Little Chalfont, UK). Selected aliquots were stained overnight at 4°C with 120 μg ml^−1^ Cy3-labeled non-specific anti-phosphotyrosine pY72 antibody (generous gift of Peter Parker CRUK or commercially Abcam Plc, Cambridge, UK) in the blocking buffer. Cells were finally washed twice and re-suspended in TBSE.

#### Membrane lipid order

Membrane liquid order, influenced by membrane cholesterol content, was investigated in HEK293T cells. Cholesterol was extracted from the cell membrane with methyl-β-cyclodextrin or supplemented with extrinsic cholesterol from a cholesterol/cyclodextrin solution. Four 0.22-μm syringe filtered incubation solutions were prepared in Opti-MEM. Methyl-β-cyclodextrin (Sigma-Aldrich) solutions were prepared at concentrations of 6.8 mg ml^−1^ and 3.4 mg ml^−1^. Water-soluble cholesterol/cyclodextrin (Sigma-Aldrich) solutions were prepared saturated in Opti-MEM and diluted to 50% (vol/vol) of the saturated solution. Cells were initially rinsed twice with pre-warmed Opti-MEM and incubated for 60 min at 37°C in either Opti-MEM or one of four the incubation solutions. Cells were then lifted by trypsin/EDTA, rinsed with PBS, and incubated with a 2.5 μM solution of di-4-ANEPPDHQ in Opti-MEM for 30 min at 37°C. Following two rinses with Opti-MEM, cells were re-suspended in Opti-MEM/25 mM HEPES buffer and immediately analyzed on the microfluidic flow cytometer. During the measurement, cells were collected into dishes with culture medium and grown in the incubator for three more days to ensure their viability.

### Fluorescence Lifetime-Based FRET Efficiency Calculation

Three different approaches were used to calculate the FRET efficiency of analyzed cells that introduce increasing level of correction for sample-induced artifacts. Generally, FRET efficiency was calculated from the fluorescence lifetime of individual cells expressing one of the FRET pairs and the average fluorescence lifetime of a control donor-only GFP-expressing cells (28,68):


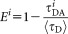
(1)


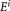
 represents the FRET efficiency of cell *i*,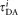 its fluorescence lifetime and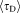 the average fluorescence lifetime of the control donor-only GFP-expressing cells.

The fluorescence lifetime in cells expressing the FRET standards correlated negatively with the fluorescence intensity. In the first approximation, this inherent artifact was corrected by calculating the FRET efficiency using the average fluorescence lifetime of a subset of control cells with comparable fluorescence intensities:


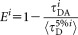
(2)


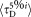
 refers to the average fluorescence lifetime of a subset of 5% of the control donor-only GFP-expressing cells with fluorescence intensities most similar to cell *i*.

FRET not only affects the fluorescence lifetime but also leads to donor fluorescence quenching (28). In the second approximation, the FRET efficiency was calculated from a subset of the control cells featuring the most similar GFP expression level rather than fluorescence intensity to compensate for the FRET quenching effect. The calculation was performed recursively:


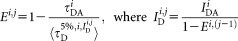
(3)

Here, FRET efficiency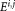 for cell *i* is derived in iteration *j* using the FRET efficiency estimated in the previous iteration *j* − 1.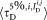 is the mean fluorescent lifetime of 5% of the control cells with FRET quenching corrected fluorescence intensity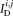 (28). The FRET efficiency was assumed to be equal to zero in the first iteration, making Eq. (3) identical to Eq. (2). The recursion was terminated once the average relative FRET efficiency change between successive iterations was less than 1%:


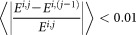
(4)

### Ratiometric FRET Efficiency Calculation

Data analysis was performed using a custom Matlab script (http://github.com/jnedbal/ratiometric-fret) according to Refs.(54), (57), (72). Single live cell populations were gated based on their scattering properties. Fluorescence signals were corrected for background autofluorescence measured in cells transfected with an empty non-fluorescent vector. Cell populations positive in all background-corrected channels were gated and subjected to mathematical analysis.

The FRET efficiency was calculated according to Refs.(54), (57), (72):


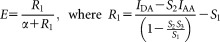
(5)

Background corrected fluorescence intensities *I*_DD_, *I*_DA_, and *I*_AA_ are the signals measured at donor emission wavelength when excited at the donor excitation (donor emission), acceptor emission wavelength when excited at the donor excitation (sensitized acceptor emission), and the acceptor emission wavelength when excited at the acceptor excitation (directly excited acceptor emission), respectively. Bleed-through correction factors *S*_1_ and *S*_3_ are derived from the GFP-only sample fluorescence intensities as a median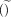 of the ratios:


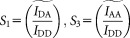
(6)

Bleed-through correction factor *S*_2_ is derived from the RFP-only sample fluorescence intensity as a median of the ratio:


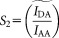
(7)

Factor *α*, essential for the determination of the FRET efficiency in Eq. (5), requires *R*_F_ to be calculated for each of the three FRET standards as follows:


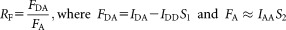
(8)

The linear regression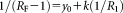 of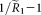 plotted against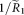 for the three FRET standards yields slope *k* and intercept *y*_0_. Factor alpha is then defined as their fraction:


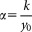
(9)

FRET efficiency is calculated from Eq. (5) using factor *α* from Eq. (9). The average, standard deviation, and standard error of the mean are calculated from the FRET efficiencies of the population excluding 10% of the outliers.

## Results

### Instrument Characterization

The instrument buildup and data analysis are described in the Methods section and in Figure 1. We characterized its performance using fluorescent particles and GFP-expressing cell lines. The instrument's response to pump pressures varying between 15 and 80 kPa was assessed. The flow rate measured by the Mitos flow rate sensor was linearly proportional to the pump pressure (Supporting Information Fig. S1a). The mean photon count remained constant (Supporting Information Fig. S1b). Burst duration and photon count were inversely proportional (Supporting Information Figs. S1c and S1d). Comparable results were obtained for 9.9 μm and 1.9 μm polystyrene fluorescent particles, 10 μm melamine fluorescent particles, and A-431 GFP-expressing cells (data not shown). The predicted throughput of the particles was estimated by the product of the particle suspension concentration and the flow rate measured using the Mitos flow rate sensor. The particle throughput measured by the instrument was calculated as the quotient of the number of bursts detected throughout the experiment and its duration. The predicted and measured throughputs were comparable for A-431 cells (Supporting Information Fig. S1e) and both sizes of the polystyrene particles (*ρ* = 1.05 g cm^−3^) (Supporting Information Figs. S1f and S1g). However, the measured throughput was roughly 20 times lower than theoretically predicted with the melamine beads (*ρ* = 1.57 g cm^−3^) (Supporting Information Fig. S1h). These experiments demonstrate predictable and reproducible operation of the flow cytometer with mammalian cell and polystyrene beads at pumping pressures between 20 and 60 kPa and throughputs reaching 3,000 particles per minute.

### Dual-Color Fluorescence Lifetime Flow Cytometry

**Figure 2:**
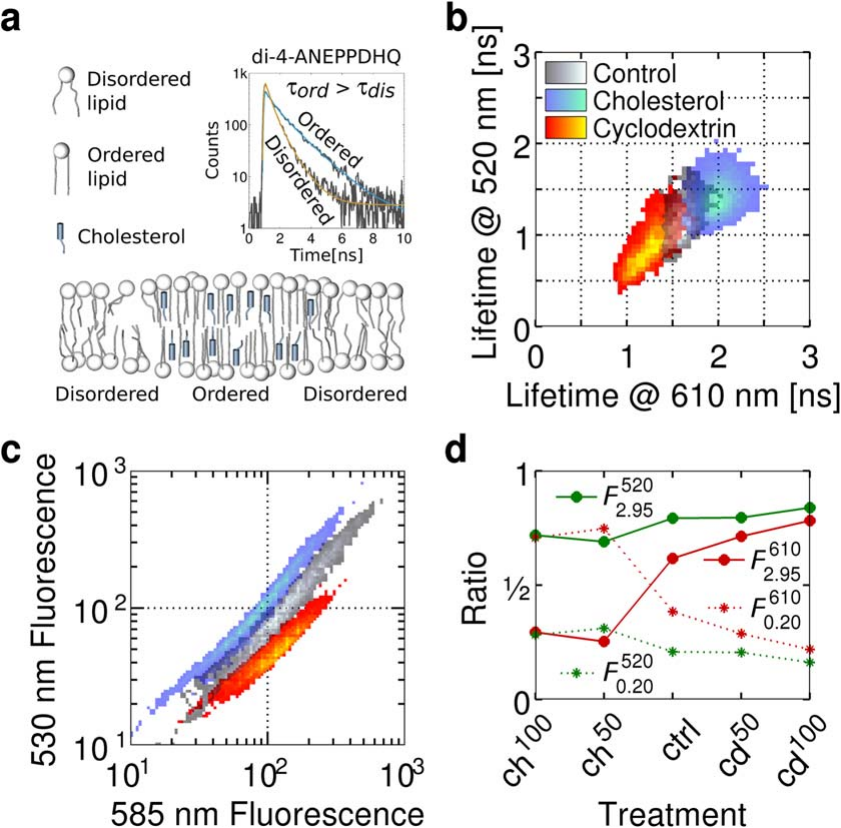
Two-color fluorescence lifetime flow cytometry. (a) Cholesterol present in cellular membranes increases their lipid order. Di-4-ANEPPDHQ is a membrane-specific dye undergoing spectral and (inset) fluorescence lifetime changes with varying lipid order. (b) Cells treated with cholesterol or cholesterol-depleting cyclodextrin increase or decrease their membrane lipid order, respectively. Di-4-ANEPPDHQ fluorescence lifetime was measured in spectral bands centered on 520 nm and 610 nm. Fluorescent lifetime progressively increased in both spectral channels with higher liquid order. (c) The same cell populations analyzed on a flow cytometer exhibit a blue shift in the emission spectrum toward shorter wavelengths (585 nm → 530 nm) with increasing membrane lipid order. (d) Cells were treated with Opti-MEM® solutions of saturated cholesterol/cyclodextrin (ch^100^), ch^100^ diluted in one-to-one volume ratio (ch^50^), Opti-MEM only as a control (ctrl) and 3.4 mg ml^−1^ (cd^50^) or 6.8 mg ml^−1^ (cd^100^) solution of methyl-β-cyclodextrin. The graph presents fractional intensities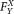 (Eq. (11)) for fluorescence lifetime components *Y* of 0.2 ns and 2.95 ns measured at wavelengths *X* of 520 nm and 610 nm for each of the five cell samples. [Color figure can be viewed in the online issue, which is available at http://wileyonlinelibrary.com.]

Multi-spectral detection capability, enabling concurrent examination of multiple biomarkers, is essential in modern flow cytometry. We added a second hybrid photomultiplier, additional spectral filters and a TCSPC router to the instrument to enable dual-color fluorescence lifetime flow cytometry. The dual-spectral channel functionality was demonstrated on cells stained with di-4-ANEPPDHQ. The fluorescence properties of di-4-ANEPPDHQ change with the degree of cellular membrane lipid packing (73,74). The dye adopts two photophysically distinct states throughout the membrane consisting of lipid ordered and lipid disordered phases (Fig. 2a). Owing to the significant overlap in the spectral components (74), at least two fluorescence lifetimes were expected across the emission band associated with these two states. Compared to the lipid ordered phase, the dye in the disordered phase should exhibit a red shift in its emission spectrum (74) and a dominant shorter fluorescence lifetime component (73,75) (Fig. 2a, inset). In accordance with the limited total number of photons emitted in a burst (typically < 10,000), the data were fitted with a mono-exponential decay, which closely resembles the average fluorescence lifetime (Fig. 2b). The proportional representation of the two assumed photophysical states of the dye was obtained through a bi-exponential global analysis of the fluorescence decays at both emission wavelengths (Fig. 2d) (69,76,77).

#### Single exponential fluorescence lifetime membrane lipid order analysis

HEK293T cell membranes were enriched or depleted of cholesterol, as described in the Methods, to alter the membrane lipid-order and thereby the fluorescent properties of di-4-ANEPPDHQ. Fluorescence lifetime measurements were performed in two spectral windows centered at 520 nm and 610 nm, corresponding to emission primarily from the lipid-ordered and lipid-disordered phases, respectively (Fig. 2b) (74). Compared to the control (untreated) cells, the fluorescence lifetime in both spectral channels decreased upon cyclodextrin treatment and increased upon cholesterol treatment. The experiment demonstrated the instruments capability to measure fluorescence lifetime at two wavelengths simultaneously.

#### Fluorescence intensity analysis of membrane lipid order

Di-4-ANEPPDHQ exhibits a spectral shift with a change in the membrane lipid order (74) that can be followed by fluorescence intensity measurements at two different wavelengths. Cells treated with cholesterol or methyl-β-cyclodextrin were analyzed on a commercial flow cytometer at two spectral windows centered at 530 nm and 585 nm (Fig. 2c). The increased prevalence of green-fluorescence emitting form of the dye (74), resulting from the increase in the membrane lipid order following cholesterol treatment, is evident from the fluorescence intensity increase at 530 nm compared to the control cells. The opposite effect of a shift from green to red fluorescence emitting form of the dye was observed in cyclodextrin-treated cells. The measured fluorescence intensity responses to the three different cell treatments were consistent with the fluorescence lifetime shifts detected in the same populations using the microfluidic flow cytometer.

#### Bi-exponential fluorescence lifetime membrane lipid order analysis

Multi-exponential fluorescence lifetime analysis offers the capability to quantify representation of different fluorophores or distinct photophysical states of a single fluorophore, such as the di-4-ANEPPDHQ. A qualitative five-step titration of cholesterol was performed, as described in the Methods, to extract the proportional representation of two photophysical dye states in cell populations with varying proportion of lipid ordered and disordered phases. Initially, two fluorescence lifetimes, most closely representing the dye states in the membrane lipid ordered and disordered phases, were established. The shorter fluorescence lifetime component measured at 520 nm in 6.8 mg ml^−1^ methyl-β-cyclodextrin treated cells represented the state of the dye inside the membrane lipid ordered phase. The longer fluorescence lifetime measured at 610 nm in cells treated with the cholesterol/cyclodextrin mixture represented the state of the dye in the membrane lipid disordered phase. The fitted fluorescence lifetimes for cells treated with cholesterol/cyclodextrin were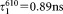 and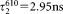 and with methyl-β-cyclodextrin were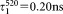 and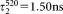. The two fluorescence lifetimes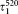 and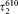 were applied in global bi-exponential analyses performed on individual cells of all five samples at both emission wavelengths according to the model represented by the simplified equation:


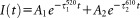
(10)

*I*(*t*) is the fluorescence decay.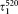 and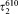, described above, represent the membrane lipid disordered and ordered phases, respectively. Amplitudes *A*_1_ and *A*_2_ are free parameters, which are fitted individually for each cell. The fractional intensities are calculated as:


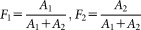
(11)

The fractional intensities for all five samples measured at 520 nm and 610 nm are presented in Figure 2d. In both spectral channels, the contribution of the longer fluorescent lifetime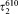 increases with decreasing cholesterol content and the subsequent rise in the proportion of the lipid disordered phase. The effect is more prominent in the 610 nm emission channel. The variation in the concentration of the cholesterol/cyclodextrin incubation solutions (ch^50^ and ch^100^ in Fig. 2d) did not cause measurable difference in the photophysical properties of the dye, presumably due to saturation of the membrane with cholesterol even at the lower concentration of the incubation solution. The results demonstrated the feasibility of bi-exponential fluorescence lifetime measurements through the application of global analysis on the data acquired with the microfluidic flow cytometer.

### HeteroFRET Validation Using FRET Standards

**Figure 3:**
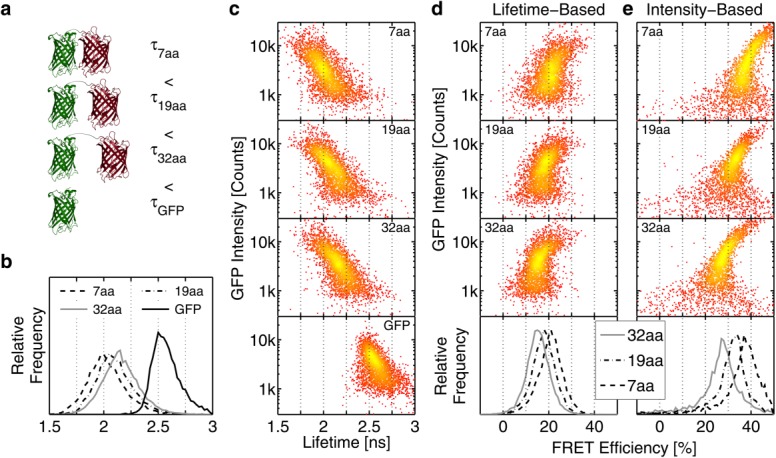
FRET in spectroscopic rulers. (a) Fluorescence lifetime and two-channel fluorescence intensity were measured in cell populations expressing GFP-RFP chimera linked by oligopeptide linkers of 7, 19, or 32 aminoacids (7aa, 19aa, 32aa) or GFP alone. (b) 1D histogram overlay compares the lifetime distributions for the four assessed cell populations. (c) From top to bottom 2D scatter plots of the GFP fluorescence intensity and fluorescence lifetime show the cell populations expressing the 7aa, 19aa, or 32aa FRET standards and the GFP. (d) 2D scatter plots of GFP fluorescence intensity versus FRET efficiency were calculated from the GFP fluorescent lifetime of cells expressing the FRET standards. Bottom panel displays a histogram overlay of the FRET efficiencies. (e) Same as (d) but calculated by the ratiometric technique based on the fluorescence intensity measurements. [Color figure can be viewed in the online issue, which is available at http://wileyonlinelibrary.com.]

FRET measurements were demonstrated on the microfluidic fluorescence lifetime flow cytometer in MCF-7 cells transiently transfected with three different FRET standards (or spectroscopic rulers). These genetically encoded molecules featured an N-terminal monomeric RFP1 (78), and a C-terminal enhanced GFP (79). GFP was the FRET donor while RFP served as the FRET acceptor. The two fluorescent protein residues were separated by linkers consisting of 32, 19, or 7 amino acids. These linkers ensured decreasing separation between fluorophores (Fig. 3a) resulting in higher resonant energy transfer efficiency and, therefore, shorter donor fluorescence lifetime. The FRET standards served as models for FRET biosensors. FRET biosensors are typically applied in the detection of the presence or concentration of specific small molecules, ions, or enzyme activity in living cells (80). FRET in biosensors can be quantified using ratiometric fluorescence intensity techniques, acceptor photobleaching, fluorescence anisotropy, or donor fluorescence lifetime. Fluorescence lifetime measurements benefit from their insensitivity to fluorophore concentration, bleaching, or variable stoichiometry and thus allow for direct comparison between experiments (38,81).

#### Fluorescence lifetime of FRET standards increases with fluorophore separation

Fluorescent lifetime was determined for transiently transfected live HEK293T cells expressing either one of the three FRET standards or GFP alone (Fig. 3a). The results of the single exponential fluorescence lifetime analysis are presented in Table 1 and Figures 3b and 3c. Consistent with the theory of FRET, the fluorescence lifetime progressively decreased with the introduction of the FRET acceptor (RFP) and shortening of the linkers separating the FRET donor (GFP) and the acceptor. A negative correlation was evident between the fluorescence lifetime and the protein expression level manifested by the GFP fluorescence intensity (Fig. 3c). The experiments demonstrated the ability to differentiate between cells expressing different FRET standards based on their fluorescence lifetime.

**Table 1:** FRET standards

	Lifetime-Based													Intensity-Based			
				FRET Efficiency (%)										FRET efficiency (%)			
	Lifetime (ns)			Equation (1)			Equation (2)			Equation (3)							
	Mean	SEM	STD	Mean	SEM	STD	Mean	SEM	STD	Mean	SEM	STD	Cells	Mean	SEM	STD	Cells
GFP-7aa-RFP	2.038	0.002	0.18	22.39	0.06	7	21.58	0.05	6	21.01	0.05	6	13,874	37.1	0.2	10	3,154
GFP-19aa-RFP	2.113	0.002	0.17	19.58	0.06	7	18.64	0.05	5	18.13	0.05	5	12,036	31.6	0.2	11	3,138
GFP-32aa-RFP	2.168	0.002	0.17	17.48	0.06	7	16.22	0.05	5	15.80	0.05	5	11,521	27.4	0.2	12	3,359
GFP	2.593	0.002	0.13										6,270				

Results of experiments with cells expressing the FRET standards GFP-7aa-RFP, GFP-19aa-RFP, GFP-32aa-RFP, and GFP only as a control are summarized here. Fluorescence lifetime, FRET efficiency, and the number of analyzed cells based on the fluorescence lifetime measurements are presented alongside the FRET efficiency and the number of analyzed cells using ratiometric fluorescence intensity measurements. The lifetime-based FRET efficiencies are results of calculations according to Eqs. (1–3), that introduce increasing levels of corrections for sample-induced artifacts. The lifetime and the FRET efficiency of each population are characterized by their mean, standard error of the mean (SEM), and standard deviation (STD). The mean values are rounded to the same decimal place as the SEM.

#### FRET efficiency in FRET standards derived from fluorescence lifetime

FRET efficiency was calculated for individual cells expressing FRET standards from their fluorescence lifetime and the average fluorescence lifetime of a GFP-expressing population using three different equations [Eqs. (1–3)]. These introduce increasing level of corrections for sample induced artifacts, stemming from the decrease of GFP fluorescence lifetime with increasing expression level. Equation (1) offers the general solution for FRET efficiency based on the fluorescence lifetime (28,68). Equation (2) corrects for the observed drop in the fluorescence lifetime of GFP with increasing expression level, based on the assumption that cells with comparable fluorescence intensities should feature comparable GFP fluorescence lifetime. Equation (3), together with the termination condition determined by Eq. (4), offer an iterative approach correcting for FRET-induced donor fluorescence quenching to calculate FRET efficiency using the assumption that cells with comparable fluorescence protein expression level should share similar GFP fluorescence lifetimes. Histograms of FRET efficiencies and their 2D scatter plots against GFP fluorescence intensities based on Eq. (3) are presented in Figure 3d. Their numerical quantification including results from all three solutions [Eqs. (1–3)] to FRET efficiency quantification are summarized in Table 1. An increase in the FRET efficiency with linker shortening and a positive correlation between the FRET efficiency and the GFP fluorescence intensity were observed across the three samples. These experiments demonstrated the feasibility of flow cytometric FRET efficiency quantification based on the use of a single control consisting of the FRET donor expressing cells.

#### FRET efficiency in FRET standards derived from fluorescence intensity

For comparison, FRET efficiency was quantified by ratiometric fluorescence intensity measurements conducted on a conventional flow cytometer with 488 nm and 561 nm excitation lasers. Two extra control samples were analyzed: cells expressing RFP only and cells transfected with an empty vector. The data analysis, principally based on Refs. (54,57,72), is described in the Methods section. Obtained FRET efficiencies are summarized in Table 1 and plotted in Figure 3e in a series of 2D scatter plots and histograms. The fluorescence lifetime-based and fluorescence intensity-based FRET efficiency estimation delivered similar positive correlation between the FRET efficiency and the GFP fluorescence intensity. However, the quantification of the FRET efficiency by the ratiometric technique gave consistently higher estimate than the fluorescence lifetime-based measurement.

### HeteroFRET Study on EGFR Tyrosine Phosphorylation

**Figure 4:**
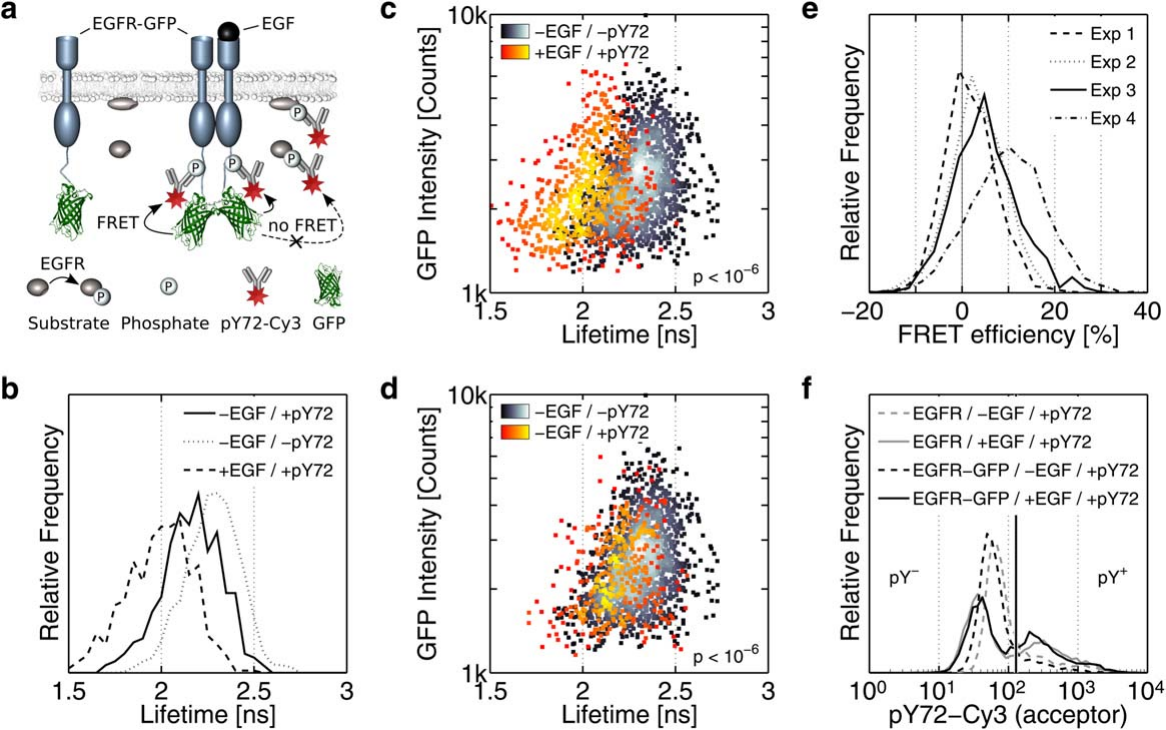
EGFR tyrosine phosphorylation in MCF-7 cells results from its kinase activity induced by the binding of a ligand such as the EGF. (a) EGF stimulation leads to GFP fluorescence lifetime decrease due to the FRET interaction between the EGFR C-terminal GFP donor (EGFR-GFP) and Cy3-tagged anti-phosphotyrosine non-specific antibody acceptor (pY72). (b) Panel shows distributions of GFP fluorescence lifetimes for the untreated cells (−EGF/−pY72), pY72-stained cells (−EGF/+pY72), and the FRET positive EGF-stimulated pY72-stained cells (+EGF/+pY72). (c, d) Data in (b) presented as 2D-scatter plots of the GFP fluorescence intensity dependence on the fluorescence lifetime. Distribution difference was assessed by a two-sample 2D Kolmogorov–Smirnov test. (e) FRET efficiency distributions in result of EGF stimulation in four independent experiments (Exp 1–4). (f) 1D histogram overlay of pY72-Cy3 antibody fluorescence intensity measured on a commercial flow cytometer demonstrates that EGF stimulation leads to a comparable increase in the proportion of phoshotyrosine positive (pY^+^) cells in populations expressing either EGFR or EGFR-GFP. [Color figure can be viewed in the online issue, which is available at http://wileyonlinelibrary.com.]

Binding of EGF leads to the up-regulation of EGFR kinase activity, resulting in tyrosine phosphorylation of EGFR itself and its downstream targets (Fig. 4a and Supporting Information Fig. S2a). Any resulting, sterically accessible, phosphorylated tyrosines can bind the pY72-Cy3 FRET acceptor antibody applied to the cells. Providing the Cy3 fluorescent residues of the antibody are within the resonant energy interaction distance of the intracellular fluorescent domain of the EGFR-GFP, FRET may occur (Fig. 4a). Consequently, the fluorescence lifetime of the GFP will decrease and the FRET efficiency increase. pY72-Cy3 being a non-specific anti-phosphotyrosine antibody, will also bind phosphorylated tyrosines on proteins other than the EGFR-GFP and thus increase the fluorescence brightness of the cell without influencing the fluorescence lifetime of the GFP. Thus, the GFP fluorescence lifetime reports on tyrosine phosphorylation occurring exclusively on the EGFR-GFP molecule.

### EGFR tyrosine phosphorylation shortens GFP lifetime

Figures 4b–4d and Table 2 present the population distributions and quantification of the GFP fluorescence lifetime, which was influenced by the FRET due to the local presence of the acceptor pY72-Cy3 antibody (Fig. 4b). The shortest fluorescence lifetime was measured in the cell population stimulated with EGF and stained with the FRET acceptor antibody pY72-Cy3. The longest fluorescence lifetime was observed in the control sample unstained with the pY72-Cy3 antibody. The control stained with the pY72-Cy3 antibody but not stimulated with EGF featured an intermediate average fluorescence lifetime resulting from quiescent EGFR phosphorylation (Supporting Information Fig. S2b) (82). The results from the same experiment were plotted as 2D scatter plots of GFP fluorescence lifetime versus intensity (Figs. 4c and 4d). The distributions from the three experimental conditions were significantly different according to a two dimensional Kolmogornov–Smirnov test against a *P*-value threshold of 10^−6^. Measuring fluorescence lifetime offers the capability to quantify EGFR tyrosine phosphorylation in response to EGF stimulation on a cell-by-cell basis.

**Table 2:** FRET in EGFR tyrosine phosphorylation

a							b				
	FRET Efficiency (%)							Lifetime (ns)			
Exp	Mean	SEM	STD	Cells	+ (%)	*τ*_GFP_ (ns)	Sample	Mean	SEM	STD	Cells
**1**	6.40	0.03	0.6	629	52	2.30	−EGF/−pY72	2.301	0.004	0.15	1,409
**2**	11.26	0.08	1.3	886	29	2.29	−EGF/+pY72	2.186	0.009	0.16	347
**3**	3.118	0.005	0.2	2,176	60	2.33	+EGF/+pY72	2.011	0.007	0.18	607
**4**	4.67	0.01	0.4	1,273	56	2.40					

(a) Table summarizes results of four repeats of the EGFR tyrosine phosphorylation experiment (Exp 1–4) (also Fig. 4e). It presents the mean FRET efficiency, the standard error of the mean (SEM), the standard deviation (STD), the number of analyzed cells, the percentage of transfected cells (+), and the lifetime of GFP without pY72-Cy3 acceptor staining (*τ*_GFP_). (b) Table presents the example results of the fluorescence lifetime analysis in Experiment 1 (also Figs.ure 4b–4d). It presents the mean fluorescence lifetime, SEM, STD, and the number of cells for the untreated control cells (−EGF/−pY72), cells stained with the pY72-Cy3 antibody (−EGF/+pY72) or the FRET-positive cells stimulated with EGF and stained with the pY72-Cy3 antibody (+EGF/+pY72). All mean values are rounded to the same decimal place as the SEM.

### EGFR tyrosine phosphorylation increases FRET efficiency

FRET efficiency was a convenient measure for comparing the EGFR tyrosine phosphorylation response to EGF stimulation in four repeats of the experiment. FRET efficiency *E^i^* for each cell *i* in the EGF-stimulated population was calculated from its respective fluorescence lifetime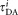 and the average lifetime of the entire control cell population unstimulated with EGF, but stained with the pY72-Cy3 antibody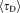 according to Eq. (1).

The four repeats of the experiment yielded FRET efficiencies summarized in Table 2 ranged between 3% and 11%. The distributions of FRET efficiencies were presented in an overlay of four histograms in Figure 4e. FRET efficiency offered a practical measure of EGFR tyrosine phosphorylation within large cell populations analyzed on the fluorescence lifetime flow cytometry.

### EGFR tyrosine phosphorylation with pY72 antibody on a conventional flow cytometer

The samples used in the aforementioned fluorescence lifetime scrutiny of EGFR tyrosine phosphorylation were subjected to analysis on a commercial flow cytometer. The EGF stimulation driving the EGFR kinase activity increased the abundance of phosphorylated tyrosines, not all of which associated with the EGFR and thus were unlikely to be involved in FRET (Fig. 4a and Supporting Information Fig. S2b). A conventional flow cytometry experiment was devised to compare the fluorescence emission from direct 488 nm excitation of Cy3 fluorescence and FRET sensitized emission from Cy3. Cells were transfected with either non-fluorescent wildtype EGFR or EGFR-GFP. Cells were then stimulated with EGF or left untreated. Results of the flow cytometry fluorescence intensity measurements with 488 nm excitation and 585 nm emission were presented in Figure 4f. A gate, based on the 99th centile of control cells transfected with an empty plasmid and stained with the pY72-Cy3 antibody, divided the population into phosphotyrosine negative (pY^−^) and phosphotyrosine positive (pY^+^) cells. FRET-sensitized emission from the pY72-Cy3 antibody due to EGFR tyrosine phosphorylation should lead to an increase in the number of pY^+^ cells and/or shift toward higher fluorescence intensities in cells expressing EGFR-GFP compared to those expressing EGFR that do not exhibit FRET. Meanwhile, the number of pY^+^ cells in EGFR-GFP expressing cells is lower (38% vs. 40%) and no measurable increase in pY72-Cy3 fluorescence was detected (Fig. 4f). The increase in the fluorescence intensity was therefore predominantly caused by the overall elevation of cellular phosphotyrosine bound by the pY72-Cy3 antibody. This increase masked the comparably lower contribution of Cy3 FRET-sensitized emission, rendering it undetectable by standard flow cytometry. Consequently, the fluorescence intensity measurements on the phosphotyrosine specific pY72 antibody cannot be used to detect EGFR phosphorylation, making the measurement of fluorescence lifetime essential.

## Discussion

### Discussion of Performed Experiments

We presented the system description and example applications of a novel microfluidic TCSPC-based fluorescence lifetime measuring flow cytometer. Its performance was characterized and operational limits were determined using test samples consisting of fluorescent particles. The feasibility of two color bi-exponential fluorescence decay measurements was demonstrated on di-4-ANEPPDHQ-stained cells with variable membrane cholesterol content. The fluorescence lifetime drop and the red shift in the emission spectrum correlated with the decrease in the membrane lipid order in line with the previously published results on generalized polarization (83,84). The fluorescent lifetime change reflected the variation to the relative proportion of the ordered and disordered phases, as shown previously, applying the fluorescence lifetime phasor analysis (73). As such, the disordered state featured a much reduced lifetime in both spectral channels but, since the ordered state is slightly red-shifted, a greater dynamic range was observed in the fluorescence lifetime measured in the 610 nm emission channel.

FRET quantification in live cells transiently expressing three FRET standards was performed and compared to ratiometric FRET analysis performed on data acquired with a conventional flow cytometer. The experiments demonstrated the technique's capacity to measure FRET efficiency in live cells, requiring only a single control consisting of cells expressing the FRET donor alone. The highly heterogeneous transiently transfected cells exhibited an artifact consisting of GFP fluorescence lifetime decrease with rising fluorescent protein expression level. Therefore three different approaches to FRET efficiency calculation were devised that introduced varying extent of correction of this phenomenon. FRET efficiency was calculated on a cell-by-cell basis by applying the standard equation for the determination of FRET efficiency based on the fluorescence lifetime of cells undergoing FRET and the average fluorescence lifetime of the donor-only control cell population [Eq. (1)]. The basic correction for the GFP fluorescence lifetime decrease was achieved with Eq. (2). This involved the fluorescence lifetime of the individual FRET standard-expressing cell and the average fluorescence lifetime of a donor-only control cell subset with comparable fluorescence intensity. To further compensate for FRET-induced donor fluorescence quenching, an iterative data analysis approach was performed using a recursive Eq. (3) and termination condition determined by Eq. (4). Fluorescence lifetimes of cells with comparable protein expression levels rather than fluorescence intensities were applied in this approach. The FRET efficiency calculated in the previous iteration was used to estimate the unquenched GFP fluorescence intensity. The average fluorescence lifetime of a control cell population subset comparable to the estimated unquenched fluorescence intensity was used for the determination of a refined estimation of the FRET efficiency [Eq. (2)]. Two iterations were sufficient to achieve tolerance of ∼0.1%. Fluorescence intensity-based correction for the GFP fluorescence lifetime variation offered by Eq. (2) resulted in an estimated average FRET efficiency drop of (1.0 ± 0.2)%. Further protein expression level based correction for FRET-induced GFP fluorescence quenching introduced by Eq. (3) resulted in further (0.50 ± 0.08)% drop in the average FRET efficiency. While the standard FRET efficiency calculation (28,68) [Eq. (1)] is suitable for characterization of homogeneous cell populations, the presented data analysis approach corrected the GFP fluorescence lifetime drop artifact specific to our model samples. Deeper understanding of the underlying photophysics could be potentially acquired through the application of global fluorescence lifetime fitting routines (69,85). Comparison with ratiometric FRET efficiency quantification was possible owing to the availability of three different FRET standards featuring the same fluorescent protein pair based on the analysis procedure described in Ref.(57). The ratiometric FRET analysis reported systematically higher values, which was most likely caused by the discrepancies between the linearity assumptions of the idealized mathematical model and the actual behavior of the fluorophores inside the cells.

The cells displayed variability in the FRET standard expression spanning two orders of magnitude. An increase in the expression level correlated with the decrease in the measured fluorescence lifetime and consequently rise in the FRET efficiency, as observed previously by FLIM (86). The same positive correlation between the FRET efficiency and the biosensor expression level was confirmed by the ratiometric FRET analysis. The presented experiments could not explain the underlying principle(s). We speculate that the fluorescence lifetime dependence on the expression level might have been related to the associated changes in the degraded protein fraction, variability in the cellular protein distribution affecting the fluorescence lifetime, or possibly intermolecular resonant transfer if the local fluorescent protein concentration reached sufficiently high level. Cells expressing low and high levels of the FRET standards, measured at the same count rate by adjusting the excitation laser intensity, yielded different fluorescent lifetimes (data not shown). Therefore, pulse pile-up could be ruled out as the leading cause for the expression level-dependent fluorescence lifetime variation. Supporting this conclusion, the ratiometric technique, insensitive to pulse pile-up, gave qualitatively comparable results. The heterogeneity observed by Matthews et al. (86) in TCSPC FLIM microscopy of these FRET standards is clearly reproduced and explained by our fluorescence lifetime and ratiometric flow cytometry.

The final EGFR phosphorylation experiment validated our method against FLIM in an assay, which is broadly applicable, not only to phosphorylation of proteins such as EGFR (49–51), Akt/PBK (48), or protein kinase C α (34) but also protein ubiquitination (52) or sumoylation (53) through the choice of an antibody with the appropriate specificity. The advantage of this FRET-based approach is its sensitivity to all tyrosine phosphorylation sites within the FRET interaction range rather than just a single one offered by a site-specific monoclonal antibody. We observed variability in the FRET efficiency response to EGF stimulation between the four repeats of the experiment ranging between 3% and 11%. This was most likely caused by the differences in the efficiency of the transfection, baseline EGFR stimulation by serum constituents, or through a differential response to EGF stimulation, which is a nonlinear process involving rapid receptor degradation (Supporting Information Fig. S2a) (71). Regardless, the assay consistently detected EGFR phosphorylation increase upon EGF stimulation with orders of magnitude higher throughput than the original FLIM-based assay (49) from which it was derived.

### Fluorescence Lifetime Flow Cytometry Versus Fluorescence Lifetime Imaging

Fluorescence lifetime flow cytometry provides an increased throughput over FLIM. It simplifies the search for rare events, removes bias in the choice of imaged cells, delivers large datasets for rigorous statistical analysis, and should allow studies on dynamic changes in live cell populations. The trade-off is the loss of any information on spatial distribution of fluorescence lifetime within the cells and the limited number of photons obtainable from each cell due to their finite dwell time in the interrogation space. Fluorescence lifetime flow cytometry should be seen as a complementary technique to FLIM, in the same way conventional intensity-based flow cytometry and imaging are.

The benefits of monitoring fluorescence lifetimes rather than intensities (or anisotropy) for measurements of protein–protein interactions by FRET are well known in the field of cell imaging (38,81). Despite this, ratiometric fluorescence intensity-based techniques remain the most common approach to quantification of FRET efficiency by means of flow cytometry (54–57). Such intensity-based FRET flow cytometry is often performed only qualitatively, as quantitative analysis relies on a range of assumptions that are in many cases experimentally difficult or impossible to fulfill (54,57). Dedicated fluorescence anisotropy measuring flow cytometers were successfully used in FRET sensing (3–5,58,59). These techniques require adaptation of a laboratory flow cytometer and rely on assumptions of sophisticated models of energy transfer. In comparison, fluorescence lifetime measurement provides direct quantification of FRET efficiency based on a single control consisting of a donor only sample. Therefore, it is less prone to artifacts, more robust (38,81), and is applicable to wider range of experiments with variable FRET pair stoichiometry. Compared to ratiometric- and fluorescence anisotropy-based techniques, fluorescence lifetime flow cytometry provides a direct, robust readout with less demand for controls and applications beyond the measurement of FRET.

### Instrumental Capabilities and Outlook

The presented implementation of fluorescence lifetime flow cytometer relies on TCSPC unlike the previously published instruments measuring fluorescence lifetime by phase-modulation (9–15) or time-gating (19,20). TCSPC provides a direct measurement of fluorescence lifetime without the need for calibration. It principally allows multi-exponential fluorescence lifetime measurement, for example, to extract FRET interacting fraction (85), or in sensing applications where intraconvertable fluorescent species are present (i.e., pH (26), Ca^2+^ (27), and protein-bound NADH fraction sensing (28)). Multi-exponential fluorescence lifetime fitting requires higher photon count per particle due to increased parameter uncertainty, which could be achieved by extending the particle dwell time by decreasing the flow rate or enlarge the illumination volume, either of which would reduce the device throughput. Alternatively, global fluorescence lifetime analysis can be performed across the entire cell population (69,85,87) to extract the ratios of two or more fluorescence lifetime components for individual particles without the requirement for an increased photon count. We demonstrated the application of global analysis on the example of a two-state model of di-4-ANEPPDHQ fluorescence decay in the cellular membrane (Fig. 2d). Alternatively, the application of phasor analysis to the lifetime flow cytometry data would enable complex analysis without the need to define specific photophysical models for fitting (88). The drawbacks of TCSPC as a lifetime measurement technique in flow cytometry are the pulse pile-up effect and counting losses due to high data throughput (68). These phenomena limit the number of photons obtainable from a particle to several thousand in the case of the presented instrument. Despite these limitations the throughput of our system reaches 3,000 particles per minute, a considerable improvement over FLIM techniques. It allows gathering of a representative flow cytometry dataset in a matter of minutes. The throughput of the system and/or the photon count for each particle could be improved through the deployment of recently developed integrated TCSPC systems (89–91), which offer orders of magnitude higher maximum photon count rates over standard TCSPC through parallelization. Their application would enable accurate measurements of multi-exponential decays and even FLIM flow cytometry.
